# Conditioned media from endothelial progenitor cells cultured in simulated microgravity promote angiogenesis and bone fracture healing

**DOI:** 10.1186/s13287-020-02074-y

**Published:** 2021-01-08

**Authors:** Lingchi Kong, Yan Wang, Haixing Wang, Qi Pan, Rongtai Zuo, Shanshan Bai, Xiaoting Zhang, Wayne Yukwai Lee, Qinglin Kang, Gang Li

**Affiliations:** 1 Department of Orthopaedic Surgery, Shanghai Jiao Tong University Affiliated Sixth People’s Hospital, Yishan Rd. 600, Shanghai, 200233 People’s Republic of China; 2 Department of Orthopaedics & Traumatology, Stem Cells and Regenerative Medicine Laboratory, Li Ka Shing Institute of Health Sciences, Faculty of Medicine, The Chinese University of Hong Kong, Prince of Wales Hospital, Shatin, Hong Kong SAR PRC; 3 The CUHK-ACC Space Medicine Centre on Health Maintenance of Musculoskeletal System, The Chinese University of Hong Kong Shenzhen Research Institute, Shenzhen, People’s Republic of China; 4 Key Laboratory for Regenerative Medicine, Ministry of Education, School of Biomedical Sciences, Faculty of Medicine, The Chinese University of Hong Kong, Shatin, Hong Kong SAR PRC

**Keywords:** Endothelial progenitor cells, Microgravity, Conditioned media, Angiogenesis, Fracture healing

## Abstract

**Background:**

Paracrine signaling from endothelial progenitor cells (EPCs) is beneficial for angiogenesis and thus promotes tissue regeneration. Microgravity (MG) environment is found to facilitate the functional potentials of various stem or progenitor cells. The present study aimed to elucidate the effects of MG on pro-angiogenic properties and fracture repair capacities of conditioned media (CM) from EPCs.

**Methods:**

Human peripheral blood-derived EPCs were cultured under MG or normal gravity (NG) followed by analysis for angiogenic gene expression. Furthermore, the serum-free CM under MG (MG-CM) or NG (NG-CM) were collected, and their pro-angiogenic properties were examined in human umbilical vein endothelial cells (HUVECs). In order to investigate the effects of MG-CM on fracture healing, they were injected into the fracture gaps of rat models, and radiography, histology, and mechanical test were performed to evaluate neovascularization and fracture healing outcomes.

**Results:**

MG upregulated the expression of hypoxia-induced factor-1α (HIF-1α) and endothelial nitric oxide synthase (eNOS) and promoted NO release. Comparing to NG-CM, MG-CM significantly facilitated the proliferation, migration, and angiogenesis of HUVECs through NO-induced activation of FAK/Erk1/2-MAPK signaling pathway. In addition, MG-CM were verified to improve angiogenic activities in fracture area in a rat tibial fracture model, accelerate fracture healing, and well restore the biomechanical properties of fracture bone superior to NG-CM.

**Conclusion:**

These findings provided insight into the use of MG bioreactor to enhance the angiogenic properties of EPCs’ paracrine signals via HIF-1α/eNOS/NO axis, and the administration of MG-CM favored bone fracture repair.

**Graphical abstract:**

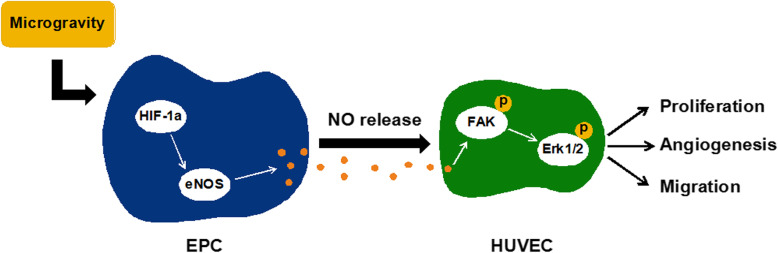

**Supplementary Information:**

The online version contains supplementary material available at 10.1186/s13287-020-02074-y.

## Background

Bone fracture caused mainly by accidents or sports injuries is a common clinical emergency [1]. Unfortunately, systemic diseases or other factor-induced delayed union or nonunion that requires repeated treatment occur in a significant proportion of this population [2, 3]. Therefore, treatment means exploration for accelerating bone regeneration is needed to relieve this predicament. Bone fracture repair is a complex process of triggering endogenous regenerative procedures to restore bone structure [1], in which neovascularization always plays crucial roles in maintaining sufficient blood supply, especially at the stage of primitive callus formation [4]. Newly formed blood vessels are in charge of transporting oxygen, nutrients, numerous cell types, and cytokines to the injury site for tissue repair. As such, the importance of modulating vascularization at fracture sites for better fracture healing is widely accepted as a new treatment option which is under extensive investigation.

Endothelial progenitor cells (EPCs) are cell populations mainly derived from bone marrow, circulate in peripheral blood, and assemble into injury site to enhance vascular repair in pathological conditions [5]. EPCs contribute to neovascularization through differentiation into mature endothelial cells [6] and paracrine manners to trigger a series of angiogenic events [7], which turns EPCs into an attractive candidate for treating stroke [8], diabetic foot [9], and traumatic bone defects [10]. However, the success of clinical application is seriously limited due to several risks including embolism formation, immunogenicity, and malignant transformation [11]. Importantly, it is reported that cell-free administration of EPCs’ derivatives including exosomes or conditioned media (CM) is as effective alternative as cell transplantation for promoting tissue revascularization and functional recovery [12–14]. There are substantial biological factors derived from EPCs including angiogenic mRNAs, microRNAs, proteins, and chemical molecules in CM. Of note, endogenous nitric oxide (NO), as a paracrine pro-angiogenic signaling molecule, is primarily synthesized by endothelial nitric oxide synthase (eNOS) and released from EPCs or mature endothelial cells during neovascularization [15, 16]. It is well known that NO could improve EPCs’ and endothelial cells’ migration, angiogenesis, and resistance to apoptosis [17–19], and therefore, NO release contributes to the paracrine properties of EPCs during tissue neovascularization.

In recent years, space flight acquires rapid development and microgravity (MG) has been reported to improve proliferation and functions of various stem or progenitor cells [20, 21]. Cardiovascular system is sensitive to gravity alteration [22, 23], and repair potentials of EPCs and endothelial cells are also influenced by MG [24, 25]. In addition, a previous study demonstrated that MG affected porcine blood-derived EPCs’ differentiation into mature endothelial cells and eNOS/NO signals in a time-dependent manner with an unclear mechanism [22]. Considering that the paracrine factors of EPCs are crucial for angiogenesis and neovascularization, we assumed that increased NO release from EPCs after MG exposure may be achieved by eNOS upregulation, and NO as an important regulator played a crucial role in endothelial cells’ angiogenesis.

The present study aimed to investigate the effects of MG on paracrine properties of EPCs and identify above hypothesis. After that, the in vitro functions of EPC-derived CM modulated by MG in angiogenesis were explored, as well as the underlying mechanisms. Finally, a rat tibial fracture model was employed to examine the in vivo effects of CM on neovascularization and fracture healing.

## Methods

### Isolation, culture, and identification of EPCs

Human peripheral blood-derived EPCs were isolated from healthy volunteers aged 18 to 30 years old as previously described [26]. Written informed consent was obtained from all of the donors, and this study was approved by the Ethics Committees of Shanghai Jiao Tong University Affiliated Sixth People’s Hospital. Briefly, peripheral blood mononuclear cells were isolated using Ficoll density gradient centrifugation according to the manufacturer’s protocol, followed by washing with phosphate-buffered saline (PBS). The cells were plated in culture dishes pre-coated with fibronectin (5 μg/cm^2^) (Merck, Darmstadt, Germany) and cultured in endothelial basal medium 2 (EBM-2; Lonza, Basel, Switzerland) supplemented with EBM-2-MV-SingleQuots (Lonza). Cells were cultured at 37 °C, 5% CO_2_ in a humidified environment, and non-adherent cells were removed after 3 days of culture. Medium was replaced every other day, and EPCs were passaged at 80–90% confluence. The cells at passage two were used in the following experiments. The morphology; cell markers including CD34, CD31, and von Willebrand factor (vWF); and angiogenic function of the remaining adherent cells were determined.

### Culture of human umbilical vein endothelial cells (HUVECs)

HUVECs were purchased from ScienCell Corporation (Shanghai, China) and cultured in Dulbecco’s modification of Eagle’s medium (DMEM; Gibco, Grand Island, NY, USA) containing 10% (v/v) fetal bovine serum (FBS; Gibco), 1% (v/v) endothelial cell growth supplements (ECGS; Gibco), and 1% (v/v) penicillin/streptomycin (P/S; Gibco) in a humidified atmosphere of 5% CO_2_ at 37 °C.

### MG simulation and CM collection

To simulate MG, we used a newly developed Gravite® (Space Bio-Laboratories Co., Ltd., Hiroshima, Japan), which produces an environment similar to that of outer space by rotating the cells around two axes and integrating the gravity vector with the temporal axis [20]. A simulated condition of 10^−3^ g was generated and monitored by a gravity acceleration sensor. EPCs grown in T25cm^2^ culture flasks were in the three-dimensional (3D) clinostat Gravite® at 37 °C in a 5% CO_2_ chamber. The air bubbles in flasks were fully evacuated to eliminate the influence of shear stress.

CM from EPCs were generated as follows: 70% confluent cells were fed with serum-free DMEM and then exposed to MG (MG-CM) or normal gravity (NG-CM) for 24 h. Cell debris was precipitated by centrifugation at 4 °C at 800×*g* for 10 min. Supernatant was collected followed by filtration with 0.22-μm filters and then stored at − 80 °C until further use.

### Chemicals

The NO scavenger Carboxy-PTIO was purchased from Beyotime Biotechnology Corporation (Shanghai, China) and worked at a concentration of 100 μM for producing NO reduced MG-CM, the NO content of which was almost equivalent to NG-CM. The Erk1/2-MAPK selective inhibitor PD98059 was purchased from Cell Signaling Technology (Danvers, MA, USA) and applied at a concentration of 50 μM.

### Cell proliferation assay

The EPCs or HUVECs were seeded in 96-well plates at an initial density of 4 × 10^3^ cells per well. DMEM, NG-CM, and MG-CM with 10% FBS were added followed by determination with 3-(4,5-dimethylthiazol-2-yl)-2,5-diphenyl tetrazolium bromide (MTT; Sigma-Aldrich, Saint Louis, MO, USA). At different time points, the cells were treated with MTT solution (0.5 mg/ml) for 4 h at 37 °C. After that, the dark blue formazan crystals formed in intact cells were solubilized with 100 μl dimethyl sulfoxide (DMSO). The absorbance was measured at 492 nm with a microplate reader.

### Quantitative real-time polymerase chain reaction (qRT-PCR)

Total RNA was extracted using Trizol reagent (Invitrogen, Carlsbad, CA, USA). Complementary DNA synthesis was performed using PrimeScript RT reagent kit (Takara, Dalian, China) according to the manufacturer’s instruction. Quantitative PCR for hypoxia-induced factor-1α (HIF-1α), eNOS, vascular endothelial growth factor (VEGF), matrix metalloproteinase-9 (MMP-9), platelet-derived growth factor-B (PDGF-B), and angiogenin-2 (Ang-2) was performed using the SYBR-Green Master Mix Plus (Applied Biosystems, Foster City, CA, USA) with ABI 7900HT System for 40 cycles. The primers were purchased from Invitrogen, and primer sequences are shown in Supplementary Table 1. The relative expression levels of genes were normalized to glyceraldehyde-3-phosphate dehydrogenase (GAPDH).

### Measurement of nitric oxide

Nitrite concentrations in the culture media were measured as an indicator of NO production using the NO detection kit (Beyotime) in accordance with the manufacturer’s protocol. Fifty microliters of medium and an equivalent amount of Griess reagent I and II were added per well. After mixing, the absorbance was measured at 540 nm with a microplate reader. NO levels in the media were calculated based on a standard curve.

### Cell migration assay

Cell migration capacity was evaluated using a 8.0-μm pore size-Transwell chambers (Corning, NY, USA). HUVECs (2 × 10^4^ cells in 200 μl DMEM or CM with 1% FBS) were loaded into the upper chamber, which was inserted into a 24-well plate with 500 μL of DMEM complete medium in the well beneath. After 12 h, cells that migrated across the transwell member were fixed with 4% paraformaldehyde (PFA; Sigma-Aldrich) and stained with 0.1% crystal violet (Solarbio, Beijing, China). The migration activity was quantified by counting the number of migrated cells under a light microscope (Nikon TE2000-E, Tokyo, Japan).

Scratch wound healing assay was also employed to determine cell migration. Briefly, HUVECs were seeded into a 6-well plate. At 100% confluence, a 100-μl pipette tip was used to make a straight scratch across the middle of each well. The cellular debris was rinsed away with PBS for twice and the cells were maintained in serum-free medium. At the time points of 0 and 12 h, photographic images of each plate were acquired under a microscope (Nikon TE2000-E). The distance migrated was assessed using Image J software.

### Tube formation assay

To perform the tube formation assay, 24-well plates were pre-coated with 150 μL Matrigel (Corning). HUVECs (1 × 10^5^ cells per well) were then seeded into the plates and cultured with serum-free medium for 6 h. The living cells were stained with Calcein-AM (2 μg/ml) (Solarbio) for 30 min at 37 °C and 5% CO_2_. After the replacement of the medium containing dye with serum-free medium, capillary-like structures were observed under a fluorescence microscope (Nikon TE2000-E) and the capacity of tube formation was quantified by calculating the total length of tubes per field using Image J software.

### Immunofluorescence staining

The HUVECs grown on cover slides were rinsed with PBS for three times and then fixed with 4% PFA for 15 min at room temperature. After the sections were treated with 0.2% (v/v) Triton X-100 in PBS for 10 min, 3% (w/v) bovine serum albumin (BSA; Sigma-Aldrich) was applied to block potential non-specific binding sites for 30 min at room temperature. The cells were then incubated with primary antibodies against Ki67 (Abcam, 1: 200, Cambridge, UK), CD31 (Abcam, 1: 200), CD34 (Abcam, 1: 150), or vWF (Abcam, 1: 200) overnight at 4 °C. After being washed, the cells were incubated with secondary antibodies (Abcam, 1: 200) coupled with TRITC or Alexa Fluor 488 for 1 h at room temperature. The slides were then mounted by using ProLongTM Gold Antifade Mountant (Invitrogen) with 4,6-diamidino-2-phenylindole (DAPI).

### Western blot analysis

Protein was extracted from cells using Radio Immunoprecipitation Assay (RIPA) lysis buffer (Sigma-Aldrich). Protein concentration was determined using the bicinchoninic acid (BCA) kit (Thermo Fisher, Waltham, MA, USA). Proteins were separated by 10% sodium dodecyl sulfate polyacrylamide gel electrophoresis (SDS-PAGE; Bio-Rad, Hercules, CA, USA) and then transferred onto polyvinylidene difluoride (PVDF) membrane (Bio-Rad). The membrane was blocked with 5% (w/v) BSA at room temperature for 1 h. Afterwards, the membrane was incubated with primary antibodies against HIF-1α (Abcam, 1: 1500), eNOS (Abcam, 1: 1500), inducible nitric oxide synthase (iNOS; Cell Signaling Technology, 1: 1500), FAK (Cell Signaling Technology, 1: 2000), p-FAK (Cell Signaling Technology, 1: 1000), Erk1/2 (Cell Signaling Technology, 1: 2000), p-Erk1/2 (Cell Signaling Technology, 1: 1000)**,** and GAPDH (Santa Cruz, 1: 2000, Heidelberg, Germany) at 4 °C overnight followed by incubating with secondary antibodies (Abcam, 1: 2000) at room temperature for 1 h. Thereafter, the proteins were visualized using Immun-Star™ HRP Chemiluminescent Kit (Bio-Rad).

### Rat fracture model

All animal experiments were approved by the Animal Research Committee of Shanghai Jiao Tong University Affiliated Sixth People’s Hospital. Forty-five 12-week-old male Sprague-Dawley rats were used to generate stabilized tibial fracture models and then randomly assigned to the DMEM (control), NG-CM, or MG-CM groups (*n* = 15 per group). The animal experimental workflow was illustrated in Fig. 5a, and surgical procedures were described as follows. Each rat was operated under general anesthesia with intraperitoneal administration of xylazine (4 mg/kg) and ketamine (40 mg/kg). A monolateral external fixator (Xinzhong, Tianjin, China) was mounted to fix the potential proximal and distal segments of the right tibia after exposure. Thereafter, a transverse osteotomy was performed at the midshaft of the tibia followed by suture. Each rat received local injection of serum-free DMEM, NG-CM, or MG-CM generated by 2 × 10^5^ cells into the fracture area on days 3, 5, and 7 after surgery. The tibia specimens were harvested on week 2 (*n* = 10 per group) and week 3 (*n* = 5 per group) after surgery.

### X-ray examination

X-ray images of the fracture sites were acquired at the postoperative 4, 7, 10, and 14 days under general anesthesia. A digital X-ray machine (Faxitron, Ultra Focus 100, Tucson, AZ, USA) was used with an exposure time of 6 s and a voltage of 32 kV. The radiographic images were scored as follows [27]: 1, no apparent hard callus; 2, slight intramembranous ossifification; 3, hard callus without bridging of the fracture gap, fracture line is apparent; 4, hard callus with bridging of the fracture gap, fracture gap is noticeable; 5, unclear boundary between the newly formed hard callus and existing cortical bone; and 6, remodeling. Evaluation was performed independently by three experienced orthopedic surgeons.

### Angiography

To evaluate neovascularization of the fracture areas, cardiac Microfil (Flow Tech, Carver, MA, USA) perfusion of rats was performed under general anesthesia. The rats were placed at 4 °C for 24 h to ensure polymerization of the contrast agent. Thereafter, the tibia samples were harvested, fixed with 4% PFA at 4 °C for 24 h, decalcified with ethylene diamine tetraacetic acid (EDTA; Solarbio) for 3 weeks and subjected to micro-computed tomography (micro-CT; Scanco Medical, Bassersdorf, Switzerland) scanning. The vessel volume of fracture areas was calculated and analyzed.

### Micro-CT analysis

Ten rats in each group were sacrificed 2 weeks after surgery, and the structure within the fracture area was quantitatively assessed using the micro-CT (15-μm thickness per slide) with a voltage of 70 kV and a current of 114 mA. Three-dimensional bone structures were shown by sagittal images of the samples. Analysis for bone volume/total tissue volume (BV/TV) of fracture area of each specimen was performed.

### Four-point bending mechanical test

The biomechanical parameters of the tibia samples were assessed using a four-point bending device (H25KS, Hounsfield Test Equipment, Surrey, UK). The tibia samples were loaded in the anterior-posterior direction at a loading rate of 5 mm/min until failure. Stiffness, ultimate load, and energy to failure were analyzed by Vernier Graphical Analysis software.

### Histology and immunohistochemistry

After fixation in 4% PFA for 24 h, the tibia specimens were decalcified in 10% EDTA for 4 weeks and embedded in paraffin. Tissue sections (7-μm-thick) were sectioned along the sagittal plane for hematoxylin-eosin (H&E) and Masson’s trichrome staining. Immunohistochemistry staining was performed using primary antibodies against CD31 (10 μg/ml, R&D, Minneapolis, MN, USA), VEGFA (Abcam, 1: 200), MMP-9 (Santa Cruz, 1: 50), and osteocalcin (OCN; Santa Cruz, 1: 50) overnight at 4 °C. After incubation with secondary antibodies (Abcam, 1: 200) conjugated with horseradish peroxidase (HRP), a Dako REAL EnVision Detection System (Dako, Glostrup, Denmark) was used to detect positive areas followed by counterstaining with hematoxylin.

### Statistical analysis

All data were presented as mean ± standard deviation. Statistical analysis was performed with Student’s *t* test for two groups or one-way ANOVA followed by Turkey’s post hoc test for comparison among multiple groups using SPSS 22.0 software (IBM, Armonk, NY), and a two-tailed *P* value less than 0.05 was considered statistically significant.

## Results

### Characterization of human peripheral blood-derived EPCs

**Fig. 1:**
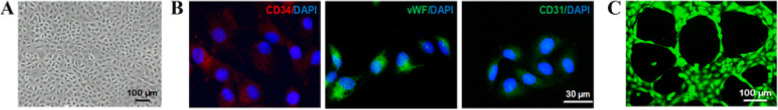
Identification of human peripheral blood-derived EPCs. **a** The isolated human peripheral blood-derived EPCs exhibited the typical endothelial-like cobblestone morphology. **b** Immunofluorescence staining revealed that the cell population was positive for CD34 (left), vWF (middle) and CD31 (right). DAPI was for nuclear counterstain. **c** Tube-like structures were generated after EPCs were seeded onto matrigel-coated dish. This experiment was repeated independently three times. Abbreviations: EPCs, endothelial progenitor cells; DAPI, 4, 6-diamidino-2-phenylindole; vWF, von Willebrand factor

After 7 days of culture, EPC-like colonies appeared and those cells within the colonies exhibited the typical cobblestone morphology of endothelial cells (Fig. 1a). The population of cells coexpressed a hematopoietic stem cell marker CD34 and endothelial markers CD31 and vWF, suggesting that these cells are endothelial and stem/progenitor lineage (Fig. 1b). In addition, biological and functional characterization demonstrated that the cells readily formed tube-like structures on seeding onto Matrigel (Fig. 1c), which is the feature of endothelial lineage cells. These results unequivocally confirmed that EPCs had been successfully isolated from the human peripheral blood.

### MG attenuated EPCs’ proliferation, but upregulated HIF-1α/eNOS/NO signal

**Fig. 2:**
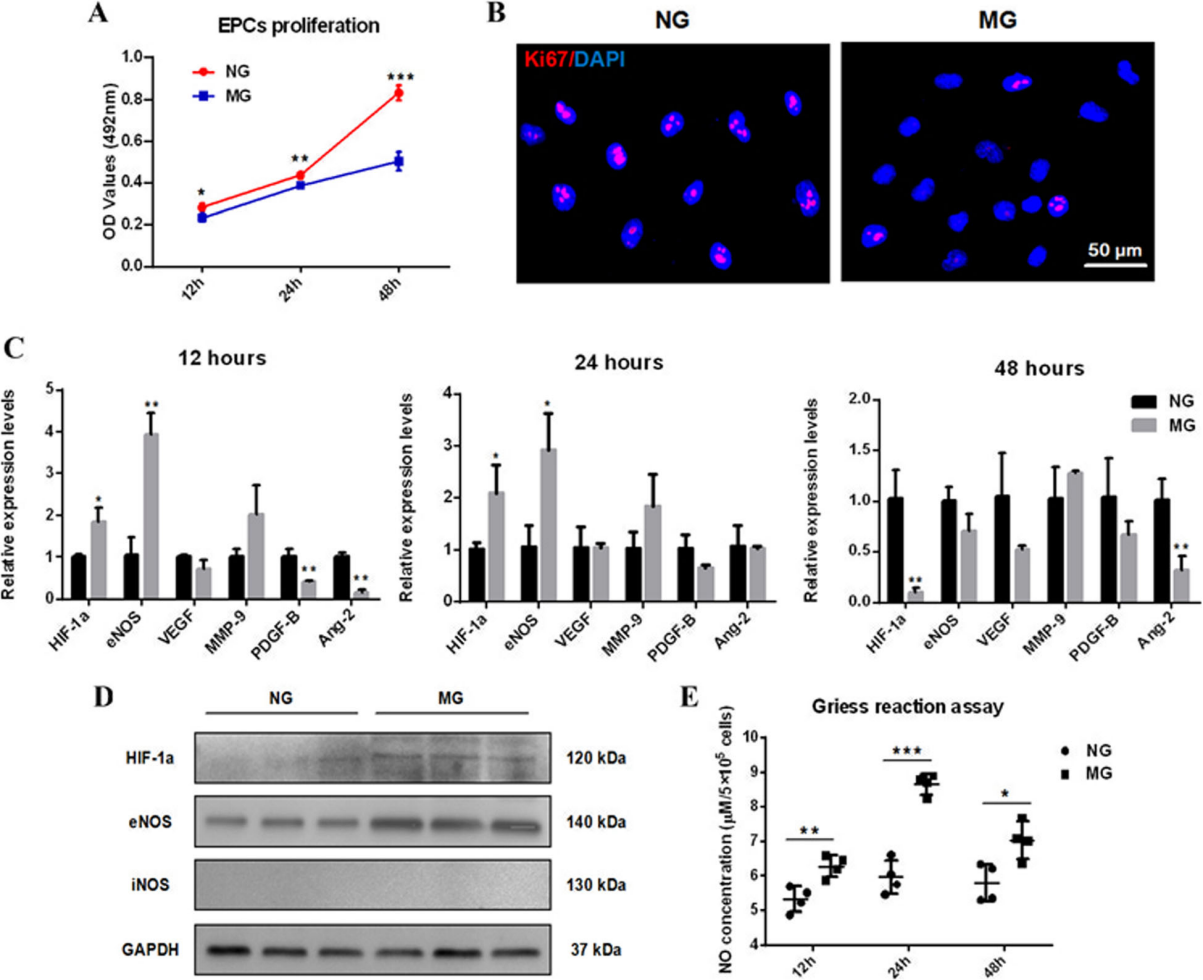
MG suppressed EPCs’ proliferation but activated HIF-1α/eNOS/NO axis. **a** MTT assay was performed to detect EPCs’ proliferation under MG or NG exposure. **b** Immunofluorescence staining showed the expression levels of Ki67 under NG (left) or MG (right) exposure. DAPI was for nuclear counterstain. **c** A cluster of angiogenic genes expression was examined by qRT-PCR after 12-, 24- and 48-h exposures of NG and MG, and GAPDH was used as the internal reference. **d** Western blot was employed to identify the expression of HIF-1α, eNOS and iNOS at protein levels. **e** Griess assay was performed to detect the NO contents in MG-CM and NG-CM after 12-, 24-, and 48-h exposures, and the concentrations were normalized to cell numbers. This experiment was repeated independently three times. Abbreviations: EPCs, endothelial progenitor cells; MG, microgravity; NG, normal gravity; DAPI, 4, 6-diamidino-2-phenylindole; GAPDH, glyceraldehyde-3-phosphate dehydrogenase; HIF-1α, hypoxia-induced factor-1α; eNOS, endothelial nitric oxide synthase; iNOS, inducible nitric oxide synthase; NO, nitric oxide; VEGF, vascular endothelial growth factor; MMP-9, matrix metalloproteinase 9; PDGF-B, platelet-derived growth factor-B; Ang-2, angiogenin-2. Data were presented as the mean ± standard deviation. *P < 0.05, **P < 0.01, and ***P < 0.001

EPCs’ proliferation cultured under MG was significantly suppressed compared to those under NG at the time points of 12 h, 24 h, and 48 h (Fig. 2a), and the immunofluorescence results indicated that the 24-h MG exposure inhibited the expression of cell proliferation marker Ki67 (Fig. 2b). The qRT-PCR detection indicated the expression of angiogenic genes HIF-1α and eNOS but VEGF, MMP-9, PDGF-B, and Ang-2 was significantly upregulated after 12- or 24-h exposure. However, the expression levels of HIF-1α and eNOS decreased as the MG exposure time prolonged (Fig. 2c). The results at protein levels were consistent to the data at the mRNA level, and iNOS was not elicited by MG (Fig. 2d). Consequently, the NO abundance in CM modulated by MG was higher than that in control media at the time points of 12 h, 24 h, and 48 h (Fig. 2e).

### MG-CM facilitated HUVECs’ proliferation, migration, and angiogenesis in vitro

**Fig. 3:**
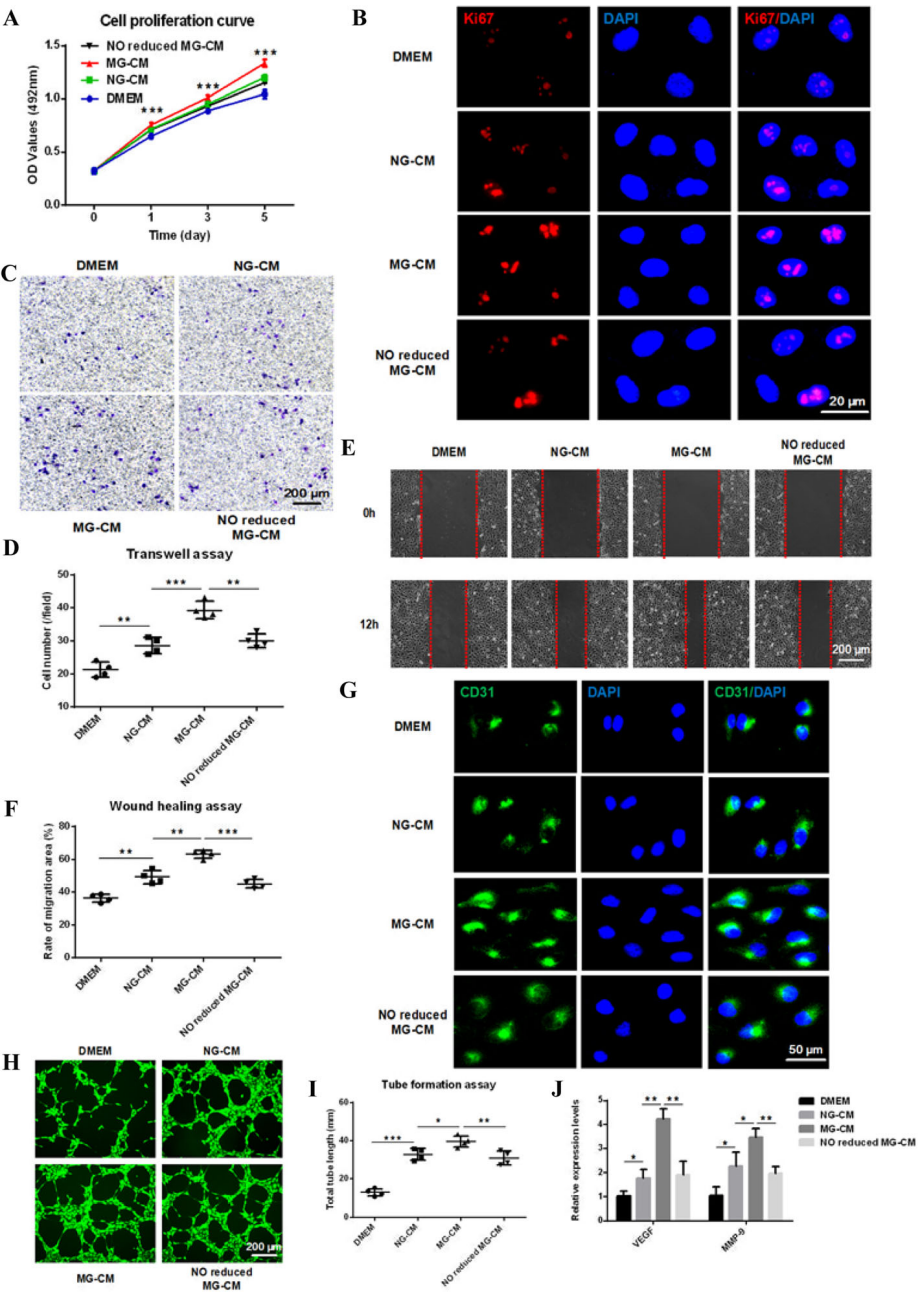
MG-CM promoted HUVECs’ proliferation, migration, and angiogenesis in vitro partially through increased NO production. **a** The effects of DMEM, NG-CM, MG-CM, and NO reduced MG-CM on HUVECs’ proliferation were analyzed by MTT assay. **b** The Ki67 expression in HUVECs was detected by immunofluorescence staining. DAPI was for nuclear counterstain. **c**–**f** Transwell assay and wound healing assay were employed to detect the migration capacity changes of HUVECs after treatment followed by quantitative analyses. **g** The CD31 expression levels of HUVECs in each group were detected by immunofluorescence staining. **h**, **i** Tube formation assay was used to detect the angiogenic abilities of HUVECs followed by quantitative analysis. **j** qRT-PCR results showed the relative expression levels of VEGF and MMP-9, with GAPDH as the internal reference. This experiment was repeated independently three times. Abbreviations: HUVECs, human umbilical vein endothelial cells; NG-CM, conditioned media from endothelial progenitor cells under normal gravity; MG-CM, conditioned media from endothelial progenitor cells under microgravity; NO, nitric oxide; DAPI, 4, 6-diamidino-2-phenylindole; GAPDH, glyceraldehyde-3-phosphate dehydrogenase; VEGF, vascular endothelial growth factor; MMP-9, matrix metalloproteinase 9. Data were presented as the mean ± standard deviation. *P < 0.05, **P < 0.01, and ***P < 0.001

The in vitro experiments were performed in HUVECs. As shown in Fig. 3a, b, MG-CM promoted the proliferation of HUVECs and the expression of Ki67 compared to NG-CM through increased NO. Consistently, the transwell assay and scratch assay confirmed that MG-CM facilitated the HUVECs’ migration in a NO-dependent manner (Fig. 3c–f), as well as CD31 expression (Fig. 3g). The matrigel tube formation assay was employed to detect the angiogenic capacities of HUVECs, and the results suggested that NO partially contributed to the enhanced angiogenic potential of MG-CM as revealed by total tube length (Fig. 3h, i). In addition, MG-CM also elevated the expression of angiogenic markers VEGF and MMP-9 in HUVECs (Fig. 3j).

### The NO-induced activation of FAK/Erk1/2-MAPK signaling pathway contributed to the enhanced pro-angiogenic effects of MG-CM

**Fig. 4:**
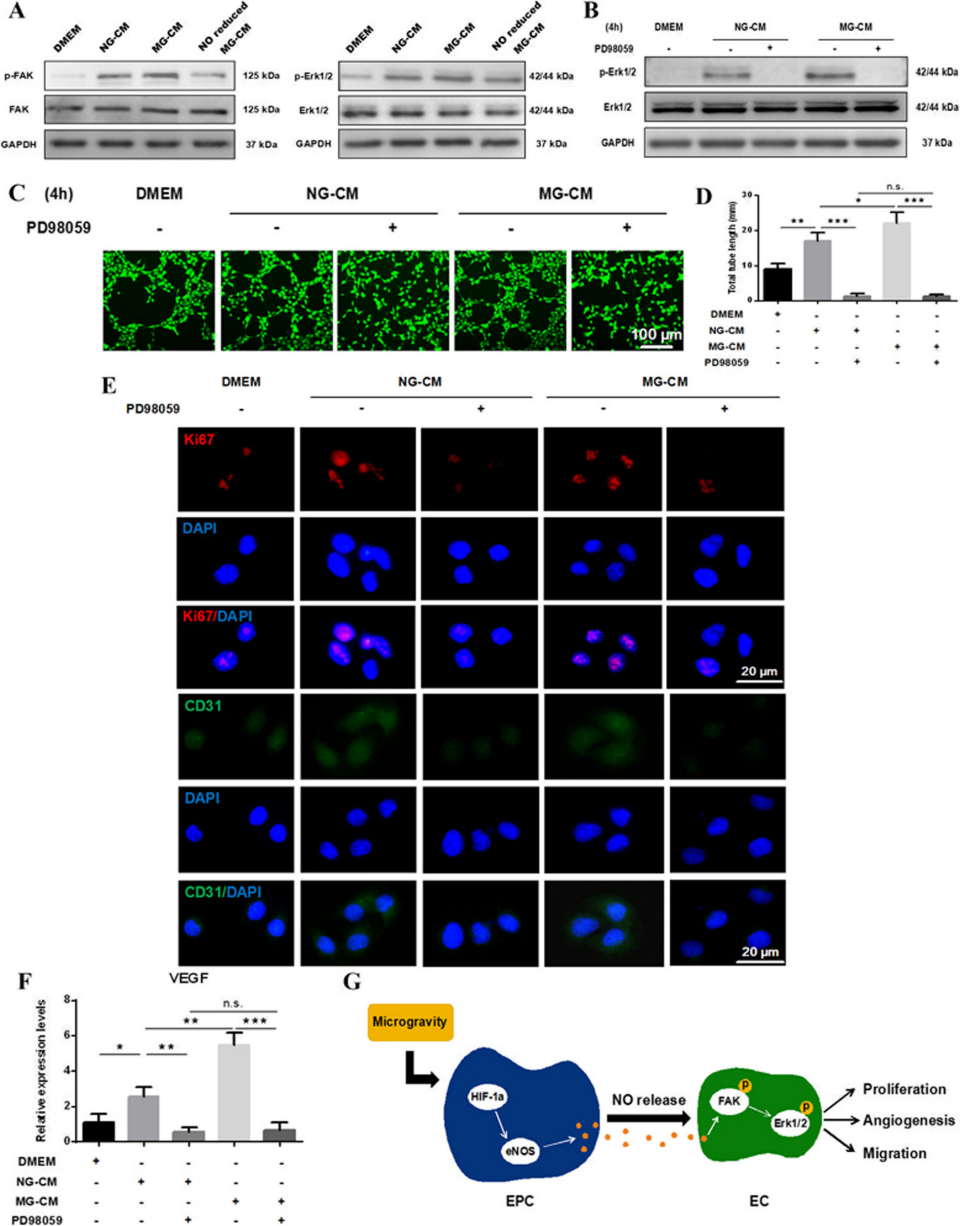
NO-induced activation of FAK/Erk1/2-MAPK signaling pathway contributed to enhanced pro-angiogenic properties of MG-CM. **a** Western blot was employed to detect the phosphorylation levels of FAK and Erk1/2. **b** The phosphorylation level of Erk1/2 was examined after Erk1/2-MAPK-selective inhibitor PD98059 application for 4 h. **c**, **d** Tube formation assay was performed to detect the angiogenic abilities of HUVECs when PD98059 was added or not, followed by quantitative analysis. **e** Immunofluorescence staining results showed the expression of Ki67 and CD31 in different groups. DAPI was for nuclear counterstain. **f** The mRNA expression level of VEGF was detected by qRT-PCR, and GAPDH was used as internal reference. **g** The schematic illustration of MG-induced HIF-1α/eNOS/NO activation of EPCs promoting HUVECs’ proliferation, migration, and angiogenesis. This experiment was repeated independently three times. Abbreviations: EPCs, endothelial progenitor cells; HUVECs, human umbilical vein endothelial cells; FAK, focal adhesion kinase; NG-CM, conditioned media from endothelial progenitor cells under normal gravity; MG-CM, conditioned media from endothelial progenitor cells under microgravity; NO, nitric oxide; DAPI, 4, 6-diamidino-2-phenylindole; GAPDH, glyceraldehyde-3-phosphate dehydrogenase; VEGF, vascular endothelial growth factor; HIF-1α, hypoxia-induced factor-1α; eNOS, endothelial nitric oxide synthase. Data were presented as the mean ± standard deviation. *P < 0.05, **P < 0.01, and *** *P* < 0.001

To investigate the underlying mechanism of MG-CM promoting angiogenesis, the phosphorylation levels of FAK and Erk1/2-MAPK were evaluated after 4-h treatment of MG-CM (Fig. 4a), and the activation of Erk1/2 could be eliminated by PD98059 (Fig. 4b), a specific inhibitor of Erk1/2-MAPK. Simultaneously, MG-CM-mediated in vitro angiogenesis and Ki67 or CD31 upregulation were also compromised when the cells were pretreated with PD98059 (Fig. 4c–e), as well as the expression of angiogenic marker VEGF (Fig. 4f), suggesting the involvement of Erk1/2-MAPK in NO-induced angiogenesis in HUVECs (Fig. 4g).

### MG-CM accelerated fracture healing and improved the mechanical properties of fracture bone in vivo

**Fig. 5:**
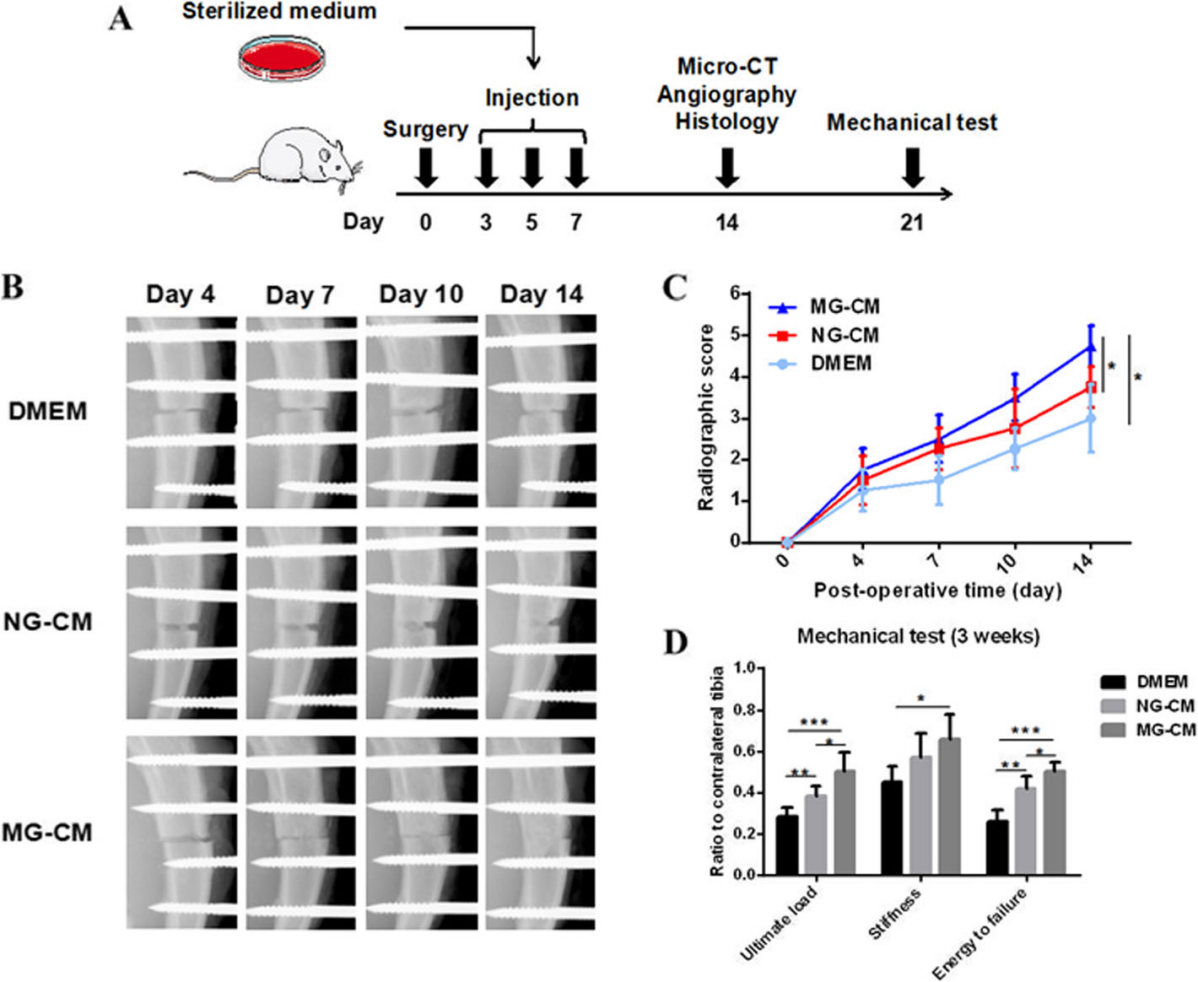
MG-CM accelerated callus growth and improved mechanical properties of fracture bone. **a** The workflow of animal experiments, including surgery, interventions, and detection. **b** The representative X-ray images of the regenerates in DMEM, NG-CM and MG-CM groups at 4, 7, 10, and 14 days after surgery. **c** The callus growth of rats was quantified by radiological scores. **d** Mechanical properties of fracture bone including ultimate load, stiffness, and energy to failure were evaluated at postoperative 3 weeks. Abbreviations: NG-CM, conditioned media from endothelial progenitor cells under normal gravity; MG-CM, conditioned media from endothelial progenitor cells under microgravity. Data were presented as the mean ± standard deviation. *P < 0.05, **P < 0.01, and ***P < 0.001

MG-CM was locally injected every other day for three times after surgery (Fig. 5a). No rat died or experienced evident complications during the experimental process. Representative tibia images of the fracture models at postoperative 4, 7, 10, and 14 days are shown in Fig. 5b. Radiographic score curves (Fig. 5c) based on these monitoring photographs revealed that both of NG-CM and MG-CM accelerated callus growth, and the latter was more prominent.

The results of mechanical assessments were normalized to the contralateral intact tibia. The values of ultimate load, stiffness, and energy to failure indicated significant improvement of mechanical properties in the MG-CM and NG-CM groups comparing to the DMEM group, and MG-CM exerted the strongest effect (Fig. 5d), suggesting a better biomechanical recovery of the suffered bone induced by MG-CM.

### MG-CM promoted neovascularization of fracture area in vivo

**Fig. 6:**
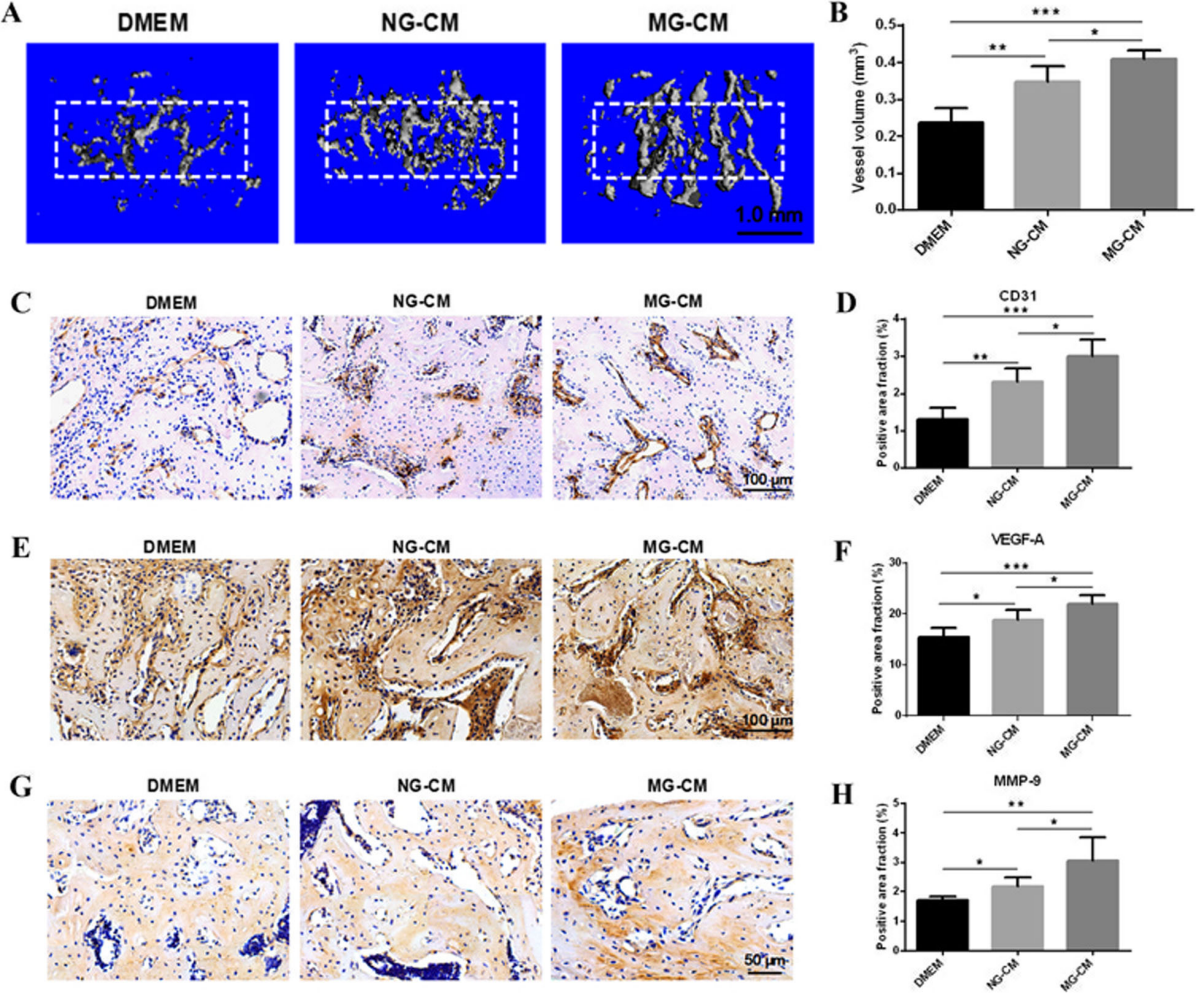
MG-CM facilitated neovascularization of fracture areas in vivo. **a** Microfil perfusion was performed to examine the newly formed vessels of the regenerated areas followed by micro-CT scan. **b** Total vessel volume was calculated and analyzed based on reconstructive vessel images. **c**–**h** Immunohistochemistry staining was employed to detect the expression of CD31, VEGF-A, and MMP-9 in regenerative areas followed by quantitative analyses. Abbreviations: NG-CM, conditioned media from endothelial progenitor cells under normal gravity; MG-CM, conditioned media from endothelial progenitor cells under microgravity; VEGF-A, vascular endothelial growth factor A; MMP-9, matrix metalloproteinase 9. Data were presented as the mean ± standard deviation. *P < 0.05, **P < 0.01, and ***P < 0.001

The results of micro-CT-based angiography demonstrated that rats with repeated injection of MG-CM exhibited more capillaries and total vessel volume when compared with DMEM and NG-CM groups (Fig. 6a, b). Immunohistochemistry staining for CD31 was performed to detect mature vessels, and VEGF-A and MMP-9 detection was employed to observe angiogenic activities. As shown in Fig. 6c–h, more CD31-positive vessels and active angiogenesis in the new bone zone in the MG-CM group than the other two groups, suggesting improved angiogenesis in fracture sites induced by MG-CM in a rat fracture model.

### MG-CM promoted fracture repair in vivo

**Fig. 7:**
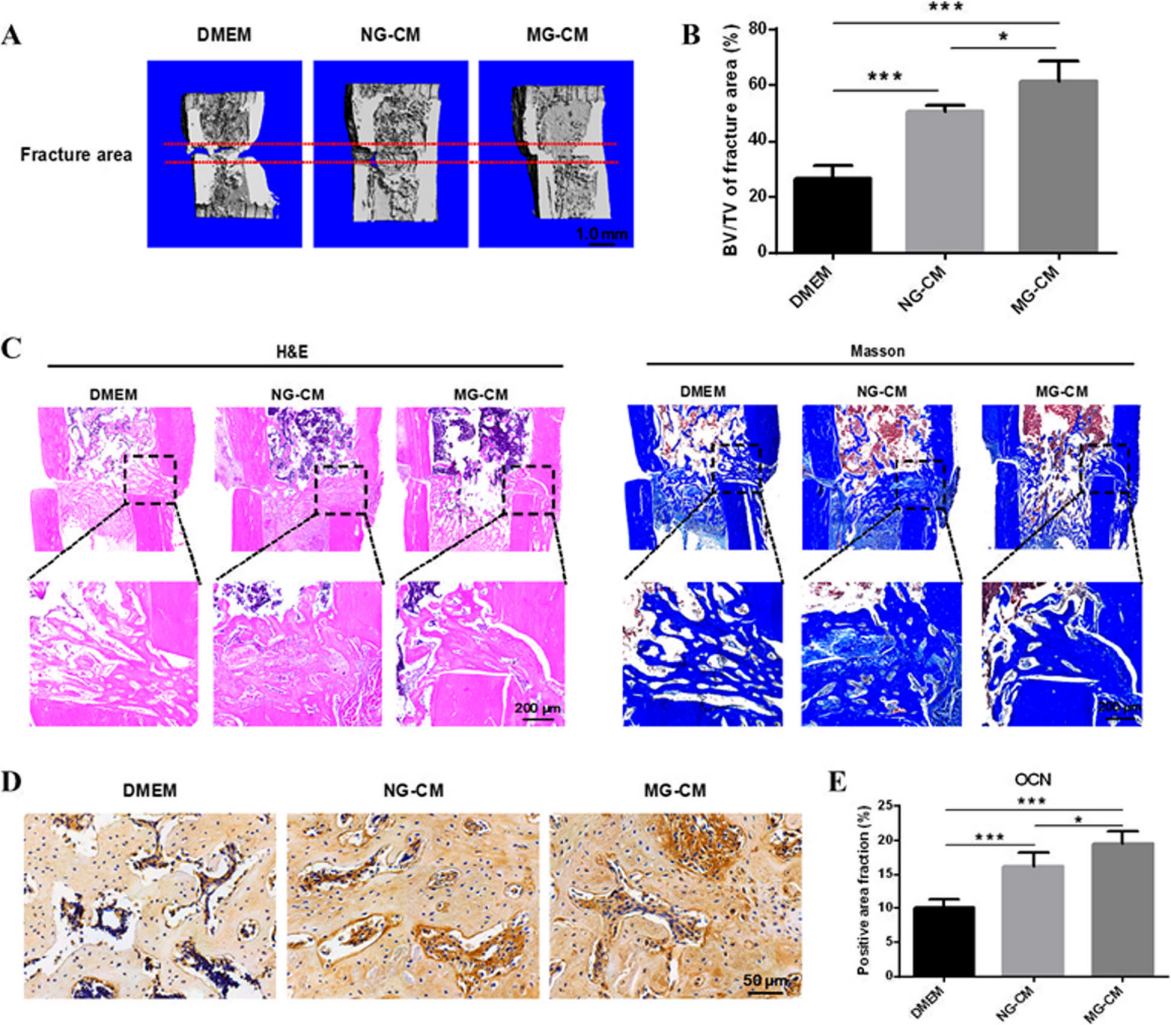
MG-CM promoted bone fracture repair in vivo. **a** The regenerative bone in fracture areas was detected by micro-CT at postoperative 2 weeks. **b** Quantitative analysis of BV/TV of regenerative tissues in fracture areas. **c** H&E staining and Masson’s trichrome staining were performed to observe the histological structures of regenerative tissues. **d**, **e** Immunohistochemistry staining was employed to detect the expression of OCN in the newly formed bone followed by quantitative analysis. Abbreviations: NG-CM, conditioned media from endothelial progenitor cells under normal gravity; MG-CM, conditioned media from endothelial progenitor cells under microgravity; BV/TV, bone volume per tissue volume; H&E, hematoxylin and eosin; OCN, osteocalcin. Data were presented as the mean ± standard deviation. *P < 0.05, **P < 0.01, and ***P < 0.001

Bone regeneration was quantified via micro-CT at 2 weeks after surgery. Sagittal planes of 3D reconstruction images of surgical tibia are shown in Fig. 7a, and the BV/TV values were significantly higher in the MG-CM group than in the DMEM and NG-CM groups (Fig. 7b), suggesting the beneficial effects of MG-CM in promoting bone regeneration. H&E and Masson’s trichrome staining of the fracture bone treated with MG-CM exhibited solid bone structure at postoperative 2 weeks in comparison with the DMEM and NG-CM groups (Fig. 7c). In DMEM-treated samples, the fracture area contained more fibrous and cartilaginous tissues and less trabecular bone near the cortical bone. Upon treatment of NG-CM or MG-CM, the fracture area displayed more mature trabecular bone tissues, and part of the callus in MG-CM group was in the process of remodeling. The OCN immunostaining showed that MG-CM application led to enhanced osteogenesis compared to control (Fig. 7d, e).

## Discussion

In this study, we found that MG activated the HIF-1α/eNOS axis and increased NO production. Although both of NG-CM and MG-CM enhanced the angiogenic capacities of endothelial cells in vitro, the MG-CM exhibited more robust potentials via NO-induced activation of FAK/Erk1/2-MAPK signaling pathway. Moreover, repeatedly local administration of MG-CM promoted neovascularization, accelerated fracture healing and improved the mechanical properties of fracture bone superior to NG-CM in a rat fracture model.

Previous studies reported that MG is a risk factor hampering cardiovascular system and the functions of endothelial cells and EPCs are regulated by gravity variations [28, 29]. Furthermore, MG alters the expression levels of microRNAs and mRNAs; those regulate mechnotransduction signals, cell migration, angiogenesis and osteogenic differentiation in cardiovascular progenitor cells [24]. Ramaswamy et al. [22] have reported that simulated MG inhibits proliferation, migration, and differentiation of the porcine blood-derived vascular stem cells, but prolonged MG exposure (more than 12 h) may restore the angiogenic capacities of this cell population. Consistently, the present study also showed that short-term MG exposure significantly attenuated the proliferation of EPCs. However, it is also reported that MG exposure followed by NG culture could improve EPCs’ expansion rates and paracrine properties [25]. Therefore, MG simulation may be a powerful bioengineering tool for eliciting cell angiogenic potentials. Previously, altered gravity was identified to regulate oxygen homeostasis and HIF-1-dependent transcripts [30, 31], and consequently refine the regenerative potentials of cells similar to hypoxic culture [32]. In our study, we for the first time found the HIF-1α/eNOS axis was activated by MG within 24 h, partially supporting the previous findings and complementing them [22, 24, 25]. As eNOS-derived NO are mainly regulated by angiogenic effectors or mechanical stress, which are responsible for vascular function maintenance, while iNOS induced NO are by proinflammatory cytokines that are usually involved in vascular pathologic processes [33, 34]. NO production in the current study was accompanied with eNOS upregulation rather than iNOS; we therefore considered that HIF-1α/eNOS axis activation mainly contributed to increased NO release. These results initially revealed the changes of NO release and its upstream signals in EPCs upon to short-term MG stimulation.

EPCs are required for neovascularization and tissue regeneration during bone fracture healing and other injury repairs [35, 36]. Furthermore, the secretomes derived from EPCs in CM such as exosomes have been demonstrated to protect endothelial function and induce angiogenesis in spinal cord injury and distraction osteogenesis [12, 37]. Exosomes derived from EPCs, as an important paracrine nanocarriers in CM, could exert the pro-angiogenic effects of EPCs by transferring RNAs, proteins, and lipids whereas avoiding the possible complications of cell transplantation including emboli formation, immunogenicity, and malignant transformation. In this study, exosomes indeed contributed to the pro-angiogenic effects of CM, and NO might also be stored in both media solution and exosomes. Since short-term MG stimulation tends to improve the paracrine properties of EPCs, we therefore evaluated the effects of MG-CM of EPCs on angiogenesis in vitro and bone fracture healing in vivo. As we hypothesized, MG-CM enhanced HUVECs’ proliferation, migration, and matrigel angiogenesis capacities, also indicated by the expression alterations of proliferation marker Ki67 and angiogenic markers CD31, VEGF, and MMP-9, while these enhanced effects were weakened to a certain extent in the presence of NO scavenger carboxy-PTIO, suggesting NO contributed, at least in part, to more robust angiogenic properties of MG-CM. Our results and previous reports [38, 39] together emphasized the pro-angiogenic effects of endogenous NO served as an active paracrine factors. In vivo, the total vessel volume and CD31 positive vessels in the regenerated areas increased after MG-CM injection as shown by angiography and histological detection, VEGF and MMP-9 expression as well. As illustrated by in vitro and in vivo data, MG-CM exhibited better pro-angiogenic capacities, which indicated that MG elicited robust angiogenic potentials of EPCs.

In terms of pro-angiogenic mechanisms, eNOS-derived NO as a well-acknowledged angiogenic gaso-transmitter regulates several key signaling pathways in cardiovascular health maintenance, including Akt-PI3K and MAPK [16, 39, 40]. Although NO is a type of gaso-transmitter, it mainly exists in CM in the form of nitrite, which is adequately stable and reversibly converted into NO to function. These biochemical properties provide a reliable prerequisite for NO to effectively activate downstream signals. FAK, a membrane-proximal tyrosine kinase, was reported to transfer NO signals to downstream Erk1/2 to promote cell survival, migration, and angiogenesis [41–44]. In this study, we also found endogenous NO in CM significantly improved endothelial cells’ proliferation, migration, and angiogenesis through stimulating tyrosine phosphorylation of FAK and its downstream Erk1/2-MAPK, while the selective inhibitor PD98059 of this pathway remarkably impaired the FAK/Erk1/2-MAPK signal transduction and pro-angiogenic effects of MG-CM. These findings suggested that NO as a crucial paracrine signal of EPCs under MG improved angiogenic capacities of endothelial cells totally or partially through FAK/Erk1/2-MAPK signaling pathway (Fig. 4g).

Neovascularization is prerequisite for the sufficient blood supply, which maintains growth of regenerative callus [45]. In our study, repeated administration of MG-CM enriched vascular network, significantly accelerating callus formation as shown by X-ray imaging and enhancing the osteogenic activities in the regenerated areas as revealed by micro-CT analyses and histological detection. In addition, mechanical properties of fracture tibia were improved in the MG-CM group comparing to the control and NG-CM groups. Therefore, the fact that robust pro-angiogenic properties of MG-CM facilitated fracture repair in this study reflected the tight couple between angiogenesis and osteogenesis although the possibility could not be excluded that MG-CM directly improved osteogenic activities. Recently, accumulating evidence [12, 46, 47] suggested that the applications of exosomes, CM, and secretome were benefit to bone regeneration, and we demonstrated that MG could further improve the fracture repair properties of CM from EPCs whereas avoiding the possible complications of cell transplantation. These findings pointed that EPCs-derived CM after MG modulation may be another useful therapeutic approach for nonunion or delayed union.

There were still limitations to this study. First, despite activation of HIF-1α/eNOS/NO, other angiogenic factors may also contribute to MG-induced enhanced angiogenic properties. Our future studies will search for additional MG-modulated angiogenic genes with proteomics and functional approaches. Second, it was reported that space flight may lead to cardiovascular harm and bone mass loss [29, 48], which seems to be contradictory with our findings in this study. Although it could be explained by distinct MG exposure duration and complicated in vivo environment, future work will be designed to fully elucidate it.

## Conclusion

The present study demonstrated that MG-induced HIF-1α/eNOS activation and increased NO production partially contributed to enhanced angiogenic properties of EPC-derived CM through FAK/Erk1/2-MAPK pathway, indicating MG tends to act as a useful bioreactor to improve paracrine angiogenic potentials of EPCs. In vivo, the local application of MG-CM promoted neovascularization and fracture healing, providing a novel bone regenerative strategy for nonunion and delayed union. Future studies are required to deep explore full-scale EPCs responses to MG and evaluate the efficacy of MG-CM in other disease models.

## Supplementary information

**Additional file 1.** Supplementary Table 1

## Data Availability

All data generated or analyzed during this study are included in this published article.

## Abbreviations

EPCs: Endothelial progenitor cells
MG: Microgravity
NG: Normal gravity
CM: Conditioned media
HIF-1α: Hypoxia-induced factor-1α
eNOS: Endothelial nitric oxide synthase
iNOS: Inducible nitric oxide synthase
HUVECs: Human umbilical vein endothelial cells
vWF: Von willebrand factor
VEGF: Vascular endothelial growth factor
MMP-9: Matrix metalloproteinase-9
PDGF-B: Platelet-derived growth factor-B
Ang-2: Angiogenin-2
OCN: Osteocalcin
GAPDH: Glyceraldehyde-3-phosphate dehydrogenase
FAK: Focal adhesion kinase
Micro-CT: Micro-computed tomography
BV/TV: Bone volume/total tissue volume
H&E: Hematoxylin-eosin
