# Supplementary material for: Conditioned media from endothelial progenitor cells cultured in simulated microgravity promote angiogenesis and bone fracture healing
Source: Stem Cell Res Ther. 2021 Jan 8;12:47. doi: 10.1186/s13287-020-02074-y (PMC7792074; doi:10.1186/s13287-020-02074-y)
Supplement: Supplementary file 1 — Additional file 1. Supplementary Table 1 [file 13287_2020_2074_MOESM1_ESM.doc]

**Table S1** The primers sequences used for qRT-PCR.

| **Genes** | **Forword primer sequence (5′-3′)** | **Reverse primer sequence(5′-3′)** |
| --- | --- | --- |
| HIF-1α | TACCCACCGCTGAAACGC | TAGGCTCAGGTGAACTTTGTCT |
| eNOS | AAGCCGCATACGCACCCAGAG | TGGGGTACCGCTGCTGGGAGG |
| VEGF | CCTGGTGGACATCTTCCAGGAGTA | CTCACCGCCTCGGCTTGTCACA |
| MMP-9 | ACGCAGACATCGTCATCCAG | CAGGGACCACAACTCGTCAT |
| PDGF-B | GCACCGAGGTGTTCGAGAT | CTGCACGTTGCGGTTGTT |
| Ang-2 | AATGCAGTACAGAACCAGACG | TTAACTTCCGCGTTTGCTCAG |
| GAPDH | TGCACCACCAACTGCTTAGC | GGCATGGACTGTGGTCATGAG |
